# Navigating the diagnostic pitfalls of cystic and solid breast masses: a comparative case report of metaplastic carcinoma and benign lesions

**DOI:** 10.3389/fonc.2026.1824750

**Published:** 2026-05-08

**Authors:** Wenjie Chu, Mengting Dong, Jiayu Sheng, Ke Jiang

**Affiliations:** Department of Breast Diseases, Yueyang Hospital of Integrated Traditional Chinese and Western Medicine, Shanghai University of Traditional Chinese Medicine, Shanghai, China

**Keywords:** cystic and solid breast mass, metaplastic breast carcinoma, benign breast tumor, breast imaging, differential diagnosis

## Abstract

**Background:**

Cystic and solid masses (CSMs) present a significant diagnostic challenge due to their heterogeneous internal architecture and a relatively high malignancy rate (23%–31%). Metaplastic breast carcinoma (MBC), an aggressive and rare subtype of triple-negative breast cancer, often presents as a CSM, frequently leading to clinical misinterpretation and delayed treatment.

**Case description:**

We report two middle-aged women presenting with rapidly enlarging, tender CSMs. Both cases exhibited similar initial clinical and sonographic features. In Case 1, the mass was initially misdiagnosed as “plasma cell mastitis” (PCM) at an external institution following a fine-needle aspiration. The definitive diagnosis of MBC was ultimately established through a vacuum-assisted breast biopsy, which was pursued after a supplementary breast magnetic resonance imaging (MRI) was obtained. In contrast, Case 2 followed a benign clinical course and was pathologically confirmed as a benign breast cyst with fibrocystic changes.

**Conclusion:**

Diagnosing CSMs requires integration of clinical history, physical examination, imaging findings, and histopathological evaluation. Pathological analysis remains the gold standard for definitive diagnosis. These cases highlight key diagnostic considerations, including the utility of supplemental breast MRI, the diagnostic value of cystic fluid characteristics, and the importance of obtaining an adequate tissue sample during biopsy.

## 1 Introduction

A cystic and solid mass (CSM) of the breast is characterized by a heterogeneous internal architecture containing both anechoic fluid and echogenic solid components. According to the Breast Imaging Reporting and Data System (BI-RADS) 6th edition, CSMs are typically categorized as BI-RADS 4, carrying a substantial malignancy risk ranging from 23% to 31% (1–3). Despite advancements in multi-modal imaging, the accurate differentiation between benign lesions and aggressive malignancies remains a formidable challenge in clinical practice. Consequently, percutaneous biopsy is mandated for a definitive diagnosis to guide subsequent management (4).

The intrinsic pathological heterogeneity of CSMs complicates the diagnostic process. Factors such as the selection of the puncture site, the amount of biopsy tissue obtained, and the proportion of cystic and solid components in actual operation may all affect the positive predictive value of suspected malignant lesions (1), increasing the risk of misdiagnosis and missed diagnosis.

This article reports the cases of two middle-aged women who presented with clinically similar rapidly growing cystic and solid breast masses but had ultimately divergent pathological outcomes. By comparing and analyzing these cases, we aim to provide clinical practitioners with valuable insights for achieving more accurate diagnosis of this type of breast lesion. Informed consent was obtained from the patients.

## 2 Case presentation

**Figure 1:**
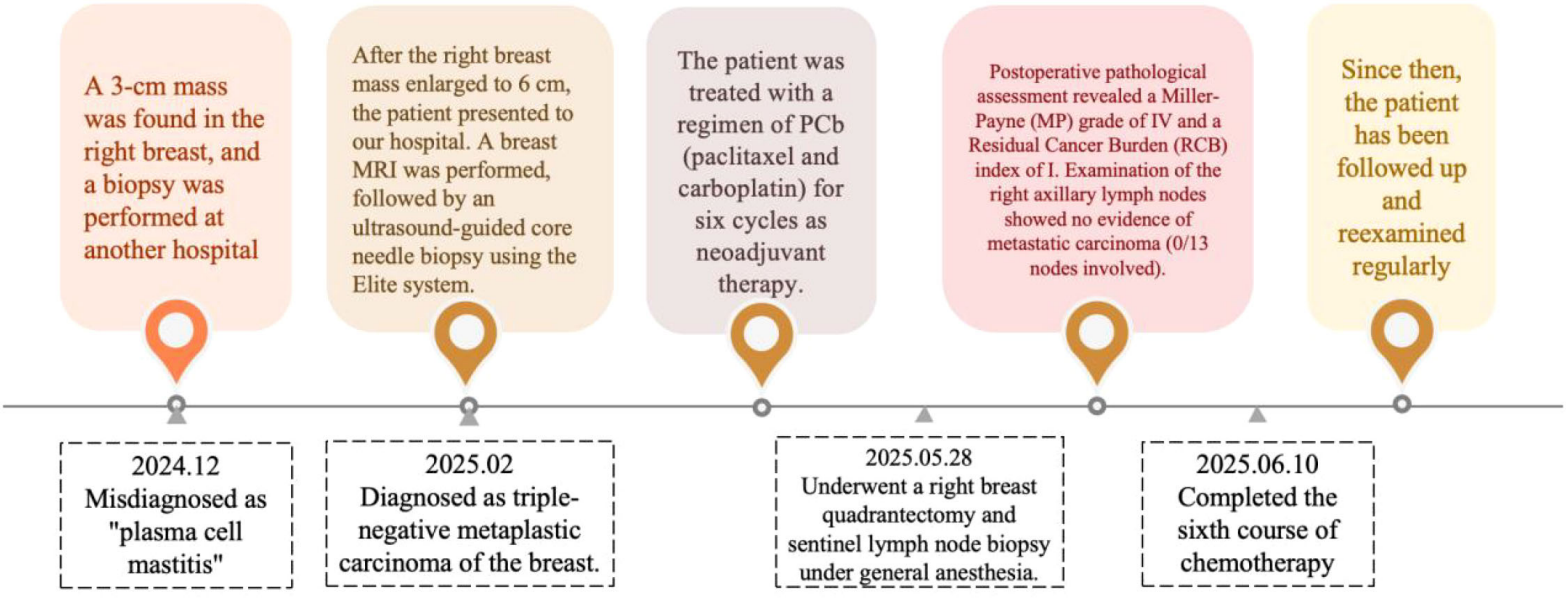
Timeline of treatment interventions (Case 1).

The timeline of the 34-year-old female patient in case 1 is shown in **Figure 1**.

**Figure 2:**
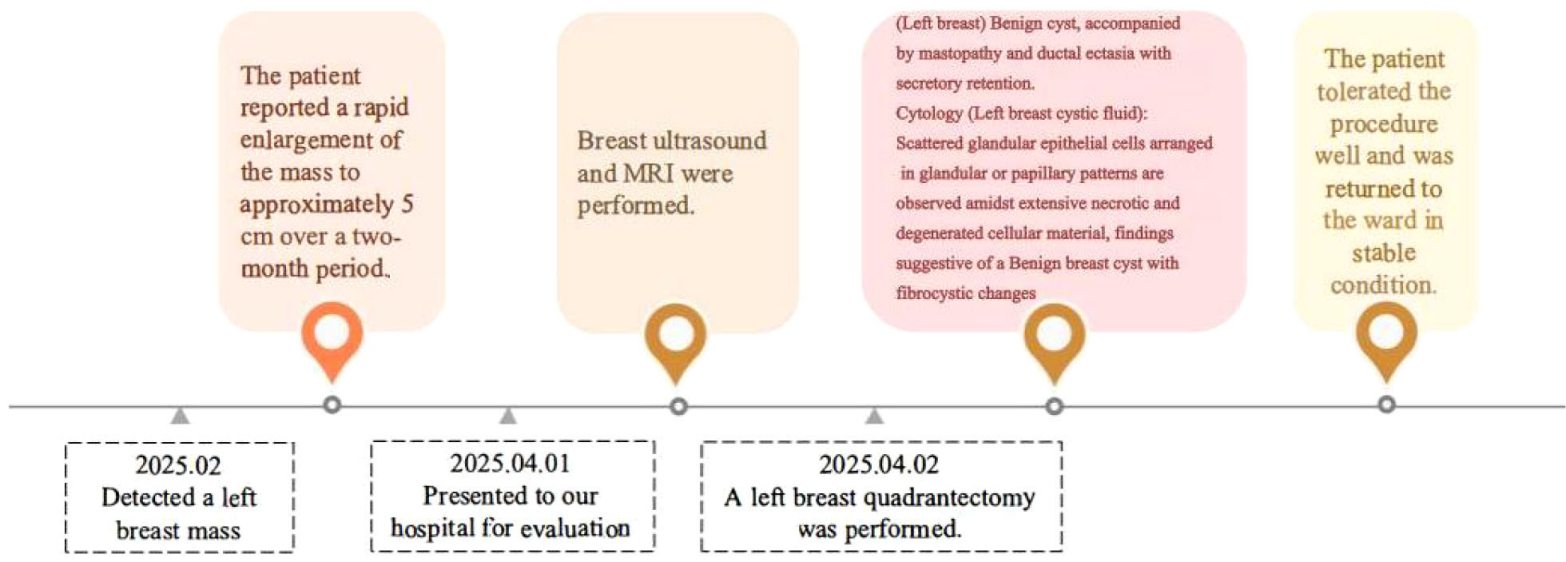
Timeline of treatment interventions (Case 2).

The timeline of the 45-year-old female patient in case 2 is shown in **Figure 2**.

## 3 Case report

### 3.1 Case 1: Metaplastic breast carcinoma

#### 3.1.1 History and initial presentation

In December 2024, a 34-year-old female incidentally identified a 3-cm mass in her right breast, accompanied by localized tenderness and mild erythema. She initially sought care at an external hospital, where a fine-needle aspiration (FNA) was performed. Histopathological analysis showed lymphocytic and plasma cell infiltration with focal ductal epithelial atypia. Although immunohistochemistry (IHC) was recommended, the patient declined. Based on the initial pathology, she was diagnosed with “plasma cell mastitis” (PCM) and prescribed oral traditional Chinese medicine (TCM). However, the lesion was refractory to treatment, and the mass rapidly progressed to 6 cm within two months. The patient subsequently presented to our institution in February 2025 for further evaluation. The patient had no family history of breast or ovarian cancer and no known genetic predispositions. She reported significant emotional stress due to the appearance of the mass. No history of smoking or alcohol use was noted.

#### 3.1.2 Physical examination

**Figure 3:**
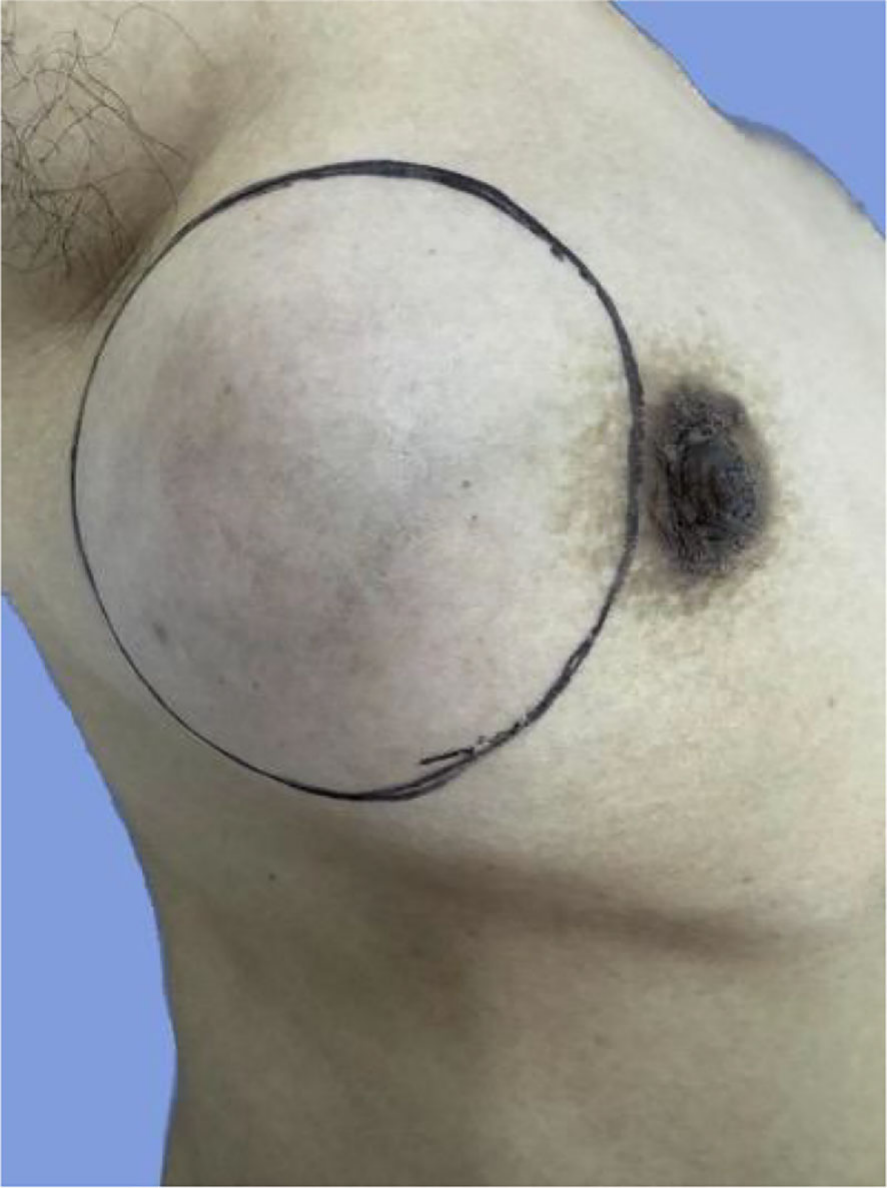
Clinical appearance of the right breast mass (Case 1). A firm, well-circumscribed, and moderately mobile mass was palpable in the outer quadrant of the right breast. The overlying skin showed erythema and localized warmth, without peau d’orange, nipple retraction, or nipple discharge.

Upon admission, physical examination revealed a firm, well-defined, and moderately mobile mass in the outer quadrant of the right breast, measuring approximately 7 cm × 6 cm. The overlying skin exhibited erythema and localized warmth, though no “peau d’orange” change, nipple retraction, or discharge was observed. The mass was not fixed to the pectoralis major muscle. Contralateral breast and bilateral axillary/supraclavicular regions showed no palpable lymphadenopathy or abnormalities (**Figure 3**).

#### 3.1.3 Imaging evaluation

**Figure 4:**
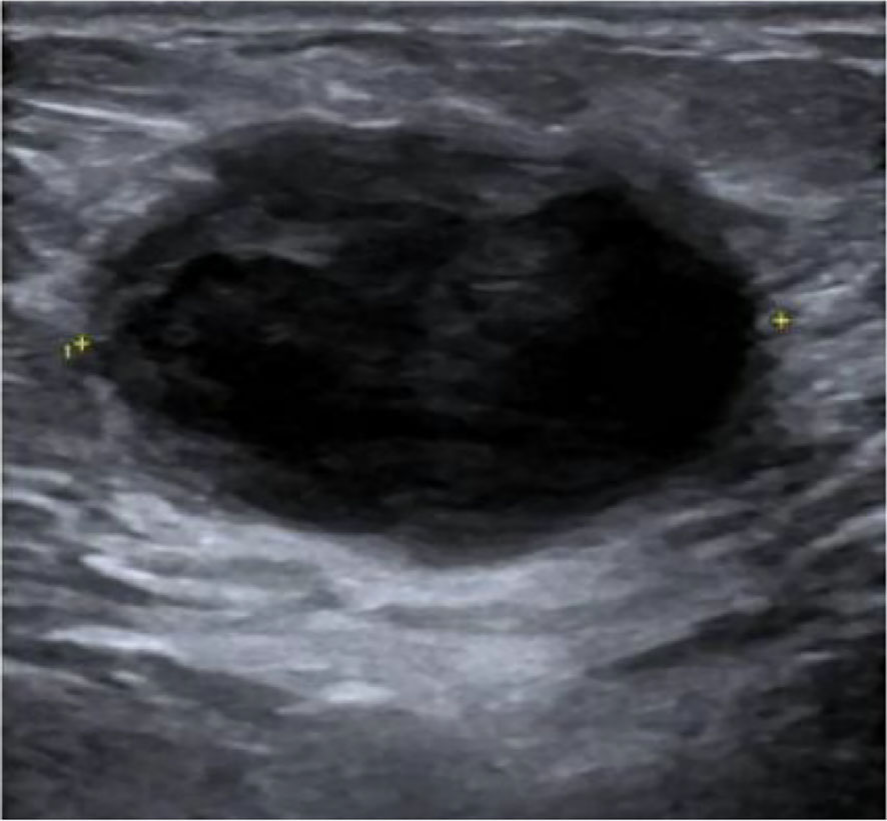
Ultrasound findings of Case 1 (February 2025). Ultrasound demonstrates a well-circumscribed cystic and solid mass (72mm×52mm×65mm) in the outer right breast. No definite internal vascularity is detected on Doppler imaging, consistent with a BI-RADS category 4A assessment.

**Figure 5:**
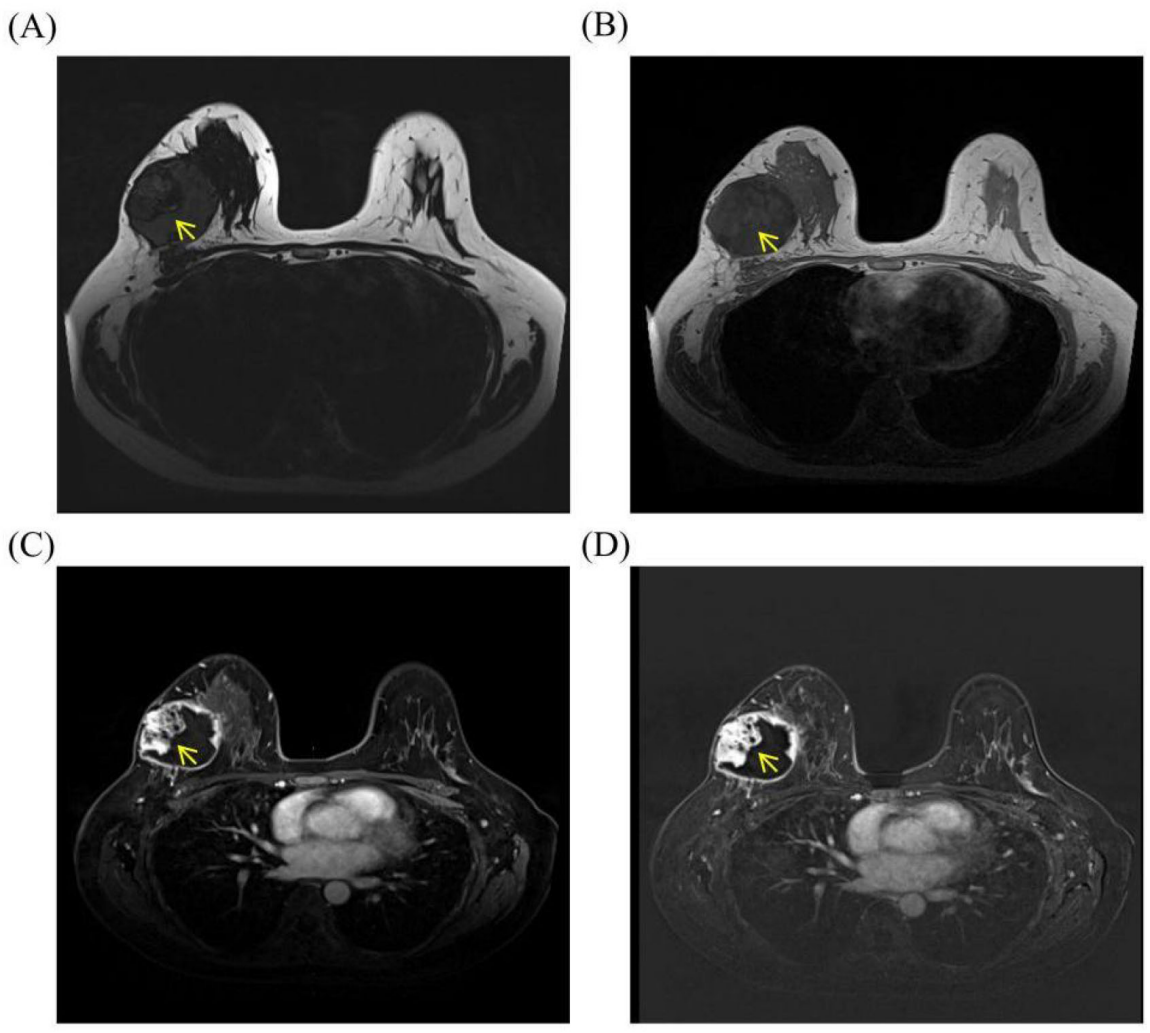
MRI findings of Case 1. **(A)** Axial T2-weighted fat-suppressed image (T2 Dixon water-only image) demonstrates a predominantly cystic mass in the upper outer and lower outer quadrants of the right breast, with an irregular inner wall, multiple mural nodules, and heterogeneous internal high- and low-signal components. **(B)** Axial pre-contrast dynamic T1-weighted image shows mural nodules along the cyst wall before contrast administration. **(C)** Axial early post-contrast dynamic image demonstrates conspicuous enhancement of the mural nodules and irregular cyst wall. **(D)** Axial subtraction image further confirms true enhancement of the mural nodules and cyst wall, supporting the presence of intracystic solid components rather than posterior background parenchymal enhancement.

Breast ultrasonography (US) identified a large, well-circumscribed CSM measuring 72mm × 52mm × 65mm. Notably, color Doppler imaging showed no internal vascularity, leading to an initial US BI-RADS 4A assessment (**Figure 4**). Due to the discordance between the rapid clinical progression and the benign US appearance, contrast-enhanced magnetic resonance imaging (MRI) was performed. MRI of the right breast demonstrated a predominantly cystic and solid mass involving the upper outer and lower outer quadrants. The lesion was characterized by an irregular inner wall, multiple mural nodules, and heterogeneous internal signal intensity on T2-weighted fat-suppressed imaging. On contrast-enhanced sequences, both the mural nodules and the cyst wall showed conspicuous enhancement, which was further confirmed on subtraction images (**Figure 5**). These findings, particularly the irregular wall morphology, multiple enhancing mural nodules, and marked enhancement pattern, were considered highly suspicious for malignancy.

(A) Axial T2-weighted fat-suppressed image (T2 Dixon water-only image) demonstrates a predominantly cystic mass in the upper outer and lower outer quadrants of the right breast, with an irregular inner wall, multiple mural nodules, and heterogeneous internal high- and low-signal components. (B) Axial pre-contrast dynamic T1-weighted image shows mural nodules along the cyst wall before contrast administration. (C) Axial early post-contrast dynamic image demonstrates conspicuous enhancement of the mural nodules and irregular cyst wall. (D) Axial subtraction image further confirms true enhancement of the mural nodules and cyst wall, supporting the presence of intracystic solid components rather than posterior background parenchymal enhancement.

#### 3.1.4 Diagnostic intervention and pathology

Given the suspicious MRI findings and the clinical history of rapid enlargement, a repeat biopsy was prioritized. To mitigate sampling bias, an ultrasound-guided vacuum-assisted breast biopsy (VABB) using the Elite system was performed. This approach ensured adequate tissue retrieval from both the solid mural nodules and the cystic walls; additionally, the cystic fluid was aspirated for liquid-based cytology.

**Figure 6:**
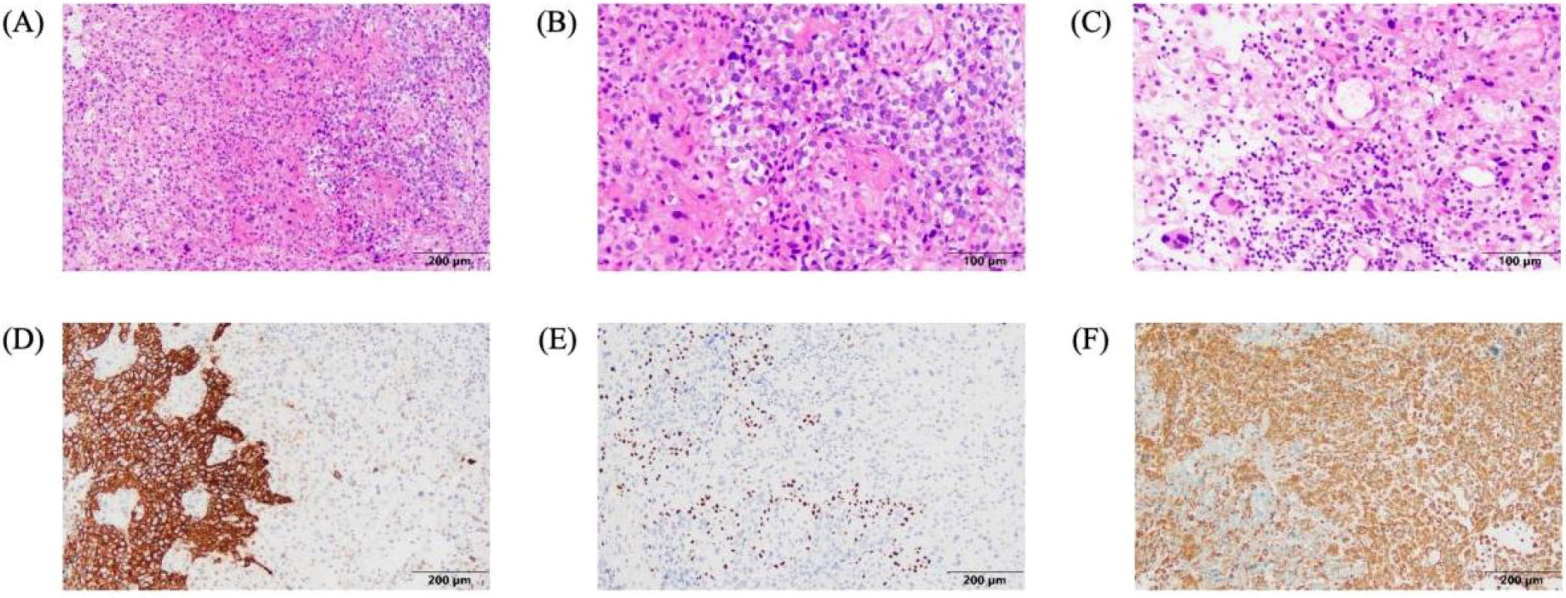
Photomicrographs of the hematoxylin and eosin (H&E)-stained sections and immunohistochemical (IHC) stains demonstrate the following histological and immunophenotypic features: **(A)** Tumor cells arranged in nests and sheets with a focally dispersed and sparse pattern (H&E, original magnification ×100). **(B)** Tumor cells with clear cytoplasm, maintaining a nested and sheet-like growth pattern (H&E, original magnification ×200). **(C)** Undifferentiated sarcomatoid component featuring scattered mononuclear and multinucleated giant cells within a loose stromal background, accompanied by interspersed lymphocytic infiltration (H&E, original magnification ×200). **(D)** The nest-forming and sheet-like tumor cells exhibit membranous and cytoplasmic immunoreactivity for CK5/6 (IHC for CK5/6, original magnification ×100). **(E)** Tumor cells at the peripheral aspects of the nests demonstrate nuclear positivity for p63 (IHC for p63, original magnification ×100). **(F)** The sarcomatoid component shows diffuse cytoplasmic positivity for vimentin (IHC for vimentin, original magnification ×100).

Pathological examination of the core tissue revealed a poorly differentiated malignant tumor consistent with MBC. IHC staining confirmed a triple-negative phenotype: ER (-), PR (-), HER-2 (-), with a high Ki-67 index of 70%. Other markers included: CK5/6 (focal +), p63 (scattered +), Vimentin (focal +), and SMA (focal +), supporting the sarcomatoid features of MBC (**Figure 6**). Liquid-based cytology also identified malignant cells, confirming the aggressive nature of the cystic component.

#### 3.1.5 Treatment and clinical outcome

The patient was diagnosed with Stage IIIA (cT3N0M0) triple-negative MBC. She underwent six cycles of neoadjuvant chemotherapy (NAC) with the PCb regimen (nab-paclitaxel 400 mg and carboplatin 800 mg) without dose reductions or treatment delays. Sequential MRI scans demonstrated a favorable response, with the tumor shrinking to 30mm × 22 mm after five cycles. Subsequently, the patient reported significant relief from breast tension and pain. On May 28, 2025, the patient underwent a right breast quadrantectomy and sentinel lymph node biopsy (SLNB). Postoperative pathology revealed a Miller-Payne grade of IV and a Residual Cancer Burden (RCB) index of I, indicating a near-complete pathological response. All 13 excised axillary lymph nodes were negative for metastasis (pN0). The patient completed the final cycle of chemotherapy on June 10, 2025. During chemotherapy, Grade 1 nausea and mild alopecia were documented as treatment-related adverse events according to the Common Terminology Criteria for Adverse Events (CTCAE), version 5.0. These adverse events were effectively controlled with standard antiemetic therapy and supportive care. No severe hematologic toxicities or abnormalities in hepatic or renal function were observed throughout the treatment course. At the 6-month follow-up, the patient reported satisfaction with the surgical outcome, improvement in quality of life, and successful resumption of daily activities.

### 3.2 Case 2: benign cystic and solid mass

#### 3.2.1 History and clinical presentation

In February 2025, a 45-year-old female self-identified a mass in her left breast. Although she initially opted for observation, the lesion demonstrated rapid interval progression, reaching approximately 5 cm within two months and causing localized tenderness. Unlike Case 1, the patient reported no associated skin erythema, edema, or local warmth. She presented to our hospital for definitive evaluation on April 1, 2025. Furthermore, the patient had no family history of breast malignancy. No significant psychological stressors or relevant genetic risk factors were identified, and she maintained a stable and healthy lifestyle.

#### 3.2.2 Physical examination

Physical examination on admission revealed a 5 cm × 4 cm palpable mass in the upper outer quadrant of the left breast. The mass was firm, well-demarcated, and exhibited moderate mobility without fixation to the pectoralis major muscle or the overlying skin. While tenderness was noted upon palpation, there were no clinical signs of malignancy, such as “peau d’orange” change or nipple retraction. The breasts remained symmetrical, and no axillary or supraclavicular lymphadenopathy was detected bilaterally.

#### 3.2.3 Imaging evaluation

**Figure 7:**
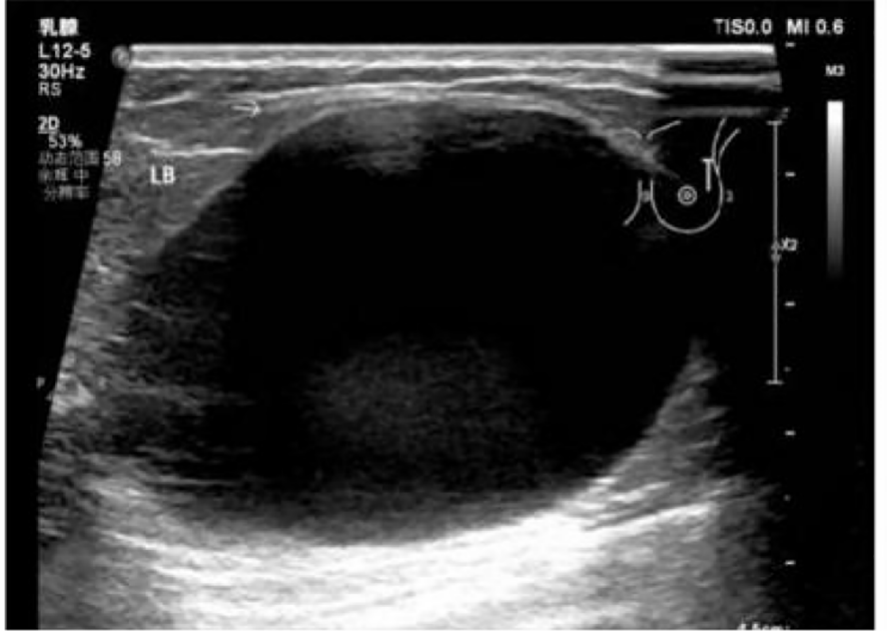
Ultrasound findings of Case 2 (April 2025). Breast ultrasonography identified a 49mm×49mm×33mm well-circumscribed cystic and solid lesion in the upper outer quadrant of the left breast. The lesion demonstrated predominantly hypoechoic echotexture with low-level internal echoes. Color Doppler imaging showed no definite internal vascularity.

**Figure 8:**
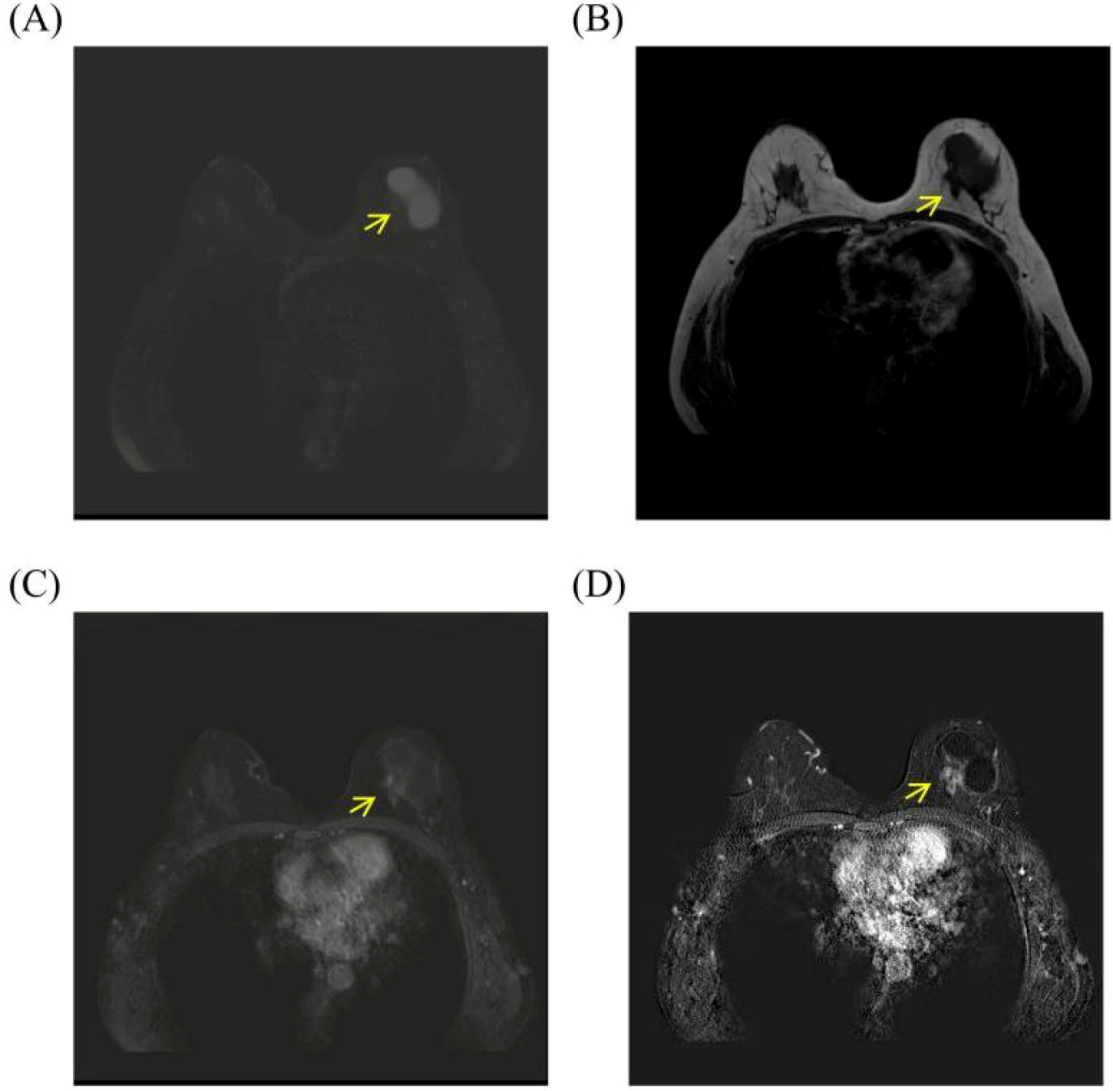
MRI findings of Case 2. **(A)** Axial T2-weighted SPAIR image shows a round cystic and solid mass in the left breast. The cystic component demonstrates marked hyperintensity, whereas the mural solid component shows intermediate signal intensity with smooth, well-circumscribed margins. **(B)** Axial T1-weighted TSE pre-contrast image shows the lesion to be isointense to slightly hypointense relative to the surrounding fibroglandular tissue. **(C)** Early dynamic contrast-enhanced eTHRIVE image demonstrates mild, relatively homogeneous enhancement of the intracystic solid component without irregular margins, spiculation, or adjacent invasive features. **(D)** Subtraction image confirms true enhancement of the mural solid component while suppressing background parenchymal signal, allowing clearer delineation of the enhancing portion of the lesion.

US identified a 49mm × 49mm × 33 mm well-circumscribed hypoechoic lesion in the left upper outer quadrant. Color Doppler imaging showed no evidence of internal vascularity, leading to an initial assessment of BI-RADS category 2–3 (**Figure 7**). To further investigate the rapidly enlarging mass, supplemental contrast-enhanced MRI was performed. The MRI demonstrated a round cystic and solid mass in the left breast. On T2-weighted fat-suppressed imaging, the cystic component showed marked hyperintensity, whereas the mural solid component exhibited intermediate signal intensity with smooth and well-circumscribed margins. On pre-contrast T1-weighted imaging, the lesion was isointense to slightly hypointense relative to the surrounding fibroglandular tissue. Following contrast administration, the mural solid component showed mild and relatively homogeneous enhancement, which was further confirmed on subtraction images. No irregular wall thickening, spiculation, or adjacent invasive features were identified (**Figure 8**). Overall, the MRI findings were more suggestive of a benign cystic and solid breast lesion.

#### 3.2.4 Surgical intervention and pathological analysis

Given the lesion’s rapid growth and the BI-RADS 4A MRI assessment, a diagnostic and therapeutic quadrantectomy was performed on April 2, 2025. Intraoperatively, the cystic component was aspirated; notably, the fluid was yellow, slightly viscous, and non-bloody—markedly distinct from the serosanguinous fluid obtained in Case 1.

**Figure 9:**
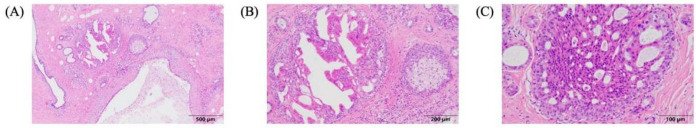
Photomicrographs of the hematoxylin and eosin (H&E)-stained sections demonstrate the following histological features: **(A)** Ductal epithelial proliferation with cysts of varying sizes (original magnification ×40). **(B)** Proliferation of ductal epithelium, featuring apocrine metaplasia with eosinophilic cytoplasm and foamy histiocytes within the glandular spaces (original magnification ×100). **(C)** Intraductal cells exhibiting florid hyperplasia with a “maturation” phenomenon, characterized by larger peripheral cells and central cells with homogeneous eosinophilic cytoplasm; the cells surrounding the cribriform spaces are arranged parallel to the luminal border (original magnification ×200).

The final histopathological diagnosis was a benign breast cyst (5 cm × 3 cm × 0.5 cm) associated with fibrocystic mastopathy and ductal ectasia with secretory retention. Liquid-based cytology of the aspirated fluid identified a few glandular epithelial cells arranged in papillary-like structures amidst necrotic debris, suggesting a benign papillary proliferation (**Figure 9**). Post-H&E staining revealed florid ductal hyperplasia with apocrine metaplasia and foamy histiocyte infiltration within the glandular spaces, but no evidence of malignancy or atypia.

#### 3.2.5 Outcome and follow-up

The perioperative period was uneventful, and the patient was discharged shortly after surgery. She expressed high satisfaction with the procedure, noting that it caused minimal disruption to her work and daily routine. No adverse or unanticipated events occurred during the clinical intervention and surgical procedures. Additionally, the patient remained asymptomatic with no evidence of recurrent lesions or complications at the 6-month post-operative follow-up.

## 4 Discussion

Owing to their heterogeneous composition, CSMs present a formidable challenge in clinico-pathological correlation. While ultrasound often serves as the initial screening tool, the overlapping features between benign inflammatory lesions and aggressive malignancies frequently lead to diagnostic ambiguity. This report contrasts two middle-aged patients with rapidly enlarging CSMs. Their divergent outcomes—one an aggressive triple-negative MBC and the other a benign cyst—underscore the necessity of a multimodal diagnostic approach.

### 4.1 Clinical similarities and differential diagnosis

MBC is a rare, high-grade malignancy that frequently presents as a CSM rather than a typical solid mass. Its rapid proliferation often outstrips the tumor’s angiogenic capacity, resulting in central ischemic necrosis and liquefaction. This pathophysiology manifests clinically as localized tenderness and skin erythema, features that closely mimic PCM.

Notably, in Case 1, the mass was refractory to TCM treatments (such as clearing heat and resolving phlegm). We emphasize that when a presumed inflammatory lesion fails to respond to conservative or TCM therapy, clinicians must immediately escalate the diagnostic workup to exclude rare malignancies like MBC.

Beyond PCM, the differential diagnosis for rapidly enlarging CSMs encompasses a broader spectrum of benign and malignant entities. Among benign lesions, breast abscess—including pyogenic abscess and that associated with granulomatous lobular mastitis (GLM)—can closely mimic an inflammatory malignancy, presenting with a thick-walled cystic structure and peripheral rim enhancement on MRI (5). Crucially, abscess tends to lack discrete mural nodules and typically demonstrates perilesional soft-tissue edema and clinical response to antibiotic therapy. Intraductal papilloma represents another important benign mimic: on MRI, papillomas predominantly display linear distribution and clumped enhancement, whereas papillary carcinoma tends toward segmental distribution with clustered ring enhancement (6). Color Doppler sonography may reveal a characteristic vascular pedicle within the solid component—a feature that suggests papillary neoplasm rather than MBC. Among malignant entities, encapsulated (intracystic) papillary carcinoma (EPC) is an indolent carcinoma that typically presents as a well-circumscribed cystic and solid mass in postmenopausal women (7). On ultrasound, EPC may appear as a circumscribed solid or cystic-solid lesion with a prominent vascular pedicle, and demonstrates intense washout enhancement on MRI. The definitive pathological distinction from MBC lies in the absence of myoepithelial cells at the tumor periphery on IHC in EPC, compared with the sarcomatoid and squamoid differentiation with basal-like markers (CK5/6, p63, vimentin) characteristic of MBC. Necrotic invasive ductal carcinoma (IDC) of no special type constitutes a further critical malignant mimic: as high-grade IDC outpaces its blood supply, central liquefactive necrosis produces a pseudocystic or CSM appearance that may be mistaken for inflammatory disease. On MRI, necrotic IDC typically exhibits thick, irregular peripheral enhancement and high-grade washout kinetics, and the irregularity and nodularity of the enhancing rim—as opposed to the smooth, thin wall of a benign cyst—serve as key discriminating features. Cystic fluid characteristics also provide important diagnostic clues: bloody or serosanguinous fluid is associated with malignancy (including MBC, EPC, and necrotic IDC), whereas clear or yellow viscous fluid, as observed in Case 2, is more consistent with benign CSM.

Due to the significant diagnostic challenges in differentiating these three conditions, a comparison of key clinical and imaging features is summarized, with histopathology serving as the gold standard for final diagnosis (**Table 1**).

**Table 1:** Differential diagnosis of CSMs: clinical and imaging features.

Features	Benign CSM				Malignant CSM		
	Periductal Mastitis (PCM)	Breast Abscess	Intraductal Papilloma (IDP)	Benign CSM (Case 2)	Metaplastic Carcinoma (Case 1)	Encapsulated Papillary Ca. (EPC)	Necrotic IDC
Clinical	Periareolar pain, heat, redness; subareolar mass; nipple retraction; no systemic fever (8)	Acute onset; fever; elevated WBC; fluctuant tender mass (9);	Bloody/serous nipple discharge (hallmark); slow-growing nodule; no skin change (10)	Palpable compressive pain;	Rapidly enlarging; erythema; skin warmth; inflammation-like; triple-negative profile (11, 12)	Slow-growing; retroareolar mass; no erythema; nipple discharge; rare (13)	Relatively rapid growth; border unclear; skin dimpling; late ‘peau d’orange’; firm palpable mass (11, 12)
US Morphology	“Tunnel sign”; dilated ducts with intraluminal debris; periductal thickening (9)	Indistinct borders; thick hypoechoic wall; internal low-level echoes; may show posterior enhancement (9)	Dilated duct with oval/lobulated solid intraluminal nodule; smooth thin wall; clear margins (10)	Circumscribed; thin smooth cystic wall; clear borders; solid mural nodule	Large cystic-solid; partially circumscribed; mural nodules; posterior acoustic enhancement (11, 12)	Circumscribed CSM; papillary projections in cyst; fibrous capsule (14, 15)	Irregular thick wall; central necrotic cavity; peripheral solid rim; cystic-solid; irregular margins (11, 12)
US Vascularity	Periductal hyperemia; high-velocity low- resistance arterial flow; no central cyst flow (9)	Peripheral annular flow; avascular abscess cavity; internal flow reduced (9)	Intraluminal vascular pedicle visible; flow in ductal wall and stalk (10)	Very sparse or absent blood flow; thin wall	Flow in solid components; avascular in necrotic areas (11, 12)	Low-resistance flow in solid component; vascularity in papillary projections (13, 14)	Peripheral thick-wall vascularity; central necrosis: avascular; high RI values (11, 12)
MRI (DCE)	Ductal zone NME; linear/segmental distribution; ductal wall enhancement (8)	Rim enhancement; T2: perilesional edema; abscess cavity: no enhancement; T2 high SI (9)	Dilated duct with intraluminal fill; NME; linear or segmental pattern (10)	Thin smooth wall; uniform cystic-solid enhancement; clear margins; no invasion	Thick irregular walls; heterogeneous peripheral enhancement; T2 high SI (necrosis/cystic degen) (11, 12)	Well-circumscribed cystic-solid; uniform enhancement of papillary projections; complete fibrous capsule on MRI (13)	Irregular thick wall; peripheral enhancement; central T2 high-signal necrosis; perilesional edema (11, 12)
Fluid Character	Green-yellow thick; “toothpaste-like” or cheesy secretion; turbid on aspiration (8)	Viscous pus; yellowish-white or gray; may contain necrotic debris; culture+ (9)	Clear serous or blood-tinged fluid; may contain RBCs; rarely frankly bloody (10)	Clear, yellow, slightly mucoid fluid	Bloody or blood- tinged; hemorrhagic necrosis content (11, 12)	Bloody fluid (13, 14)	Bloody or turbid- bloody fluid; may contain necrotic debris (11, 12)

NME, Non-mass enhancement; WBC, White blood cell; DCE, Dynamic contrast-enhanced; IDC, Invasive ductal carcinoma; IDP, Intraductal papilloma; EPC, Encapsulated papillary carcinoma; RI, Resistance index; SI, Signal intensity; RBCs, Red blood cells; CSM, Cystic and solid mass.

Superscript numbers in table cells indicate supporting references above. Case 1 and Case 2 refer to the case report entities.

### 4.2 Selection of multimodal imaging approaches

It is important to acknowledge that, according to the 2022 ACR Appropriateness Criteria for palpable breast masses, diagnostic mammography—with or without digital breast tomosynthesis—is the recommended first-line imaging modality for women aged 40 years and older, and remains appropriate for younger women when clinical suspicion is high (15). In the present report, the two patients did not undergo diagnostic mammography prior to ultrasound and MRI evaluation. This omission was contextually driven by their relatively young age and the anticipated low sensitivity of mammography in dense breast parenchyma; consequently, ultrasound combined with contrast-enhanced MRI was adopted as the primary imaging evaluation strategy. Nevertheless, we explicitly acknowledge that the absence of baseline mammographic assessment represents a methodological limitation that cannot be fully compensated by ultrasound and MRI alone, as it precludes the detection of potentially associated microcalcifications or architectural distortion that mammography may uniquely reveal. We propose that the evaluation of cystic and solid breast masses should follow a stepwise imaging approach, beginning with mammography, followed by supplemental ultrasound, and supporting MRI for selected cases when clinically indicated. This sequence is essential to prevent premature escalation to MRI before baseline mammographic characteristics have been fully assessed.

Owing to its superior soft-tissue resolution and dynamic contrast-enhanced capabilities, MRI can clearly reveal malignant characteristics. When there is a discrepancy between the clinical suspicion and initial imaging findings, supplemental contrast-enhanced MRI serves as a crucial step in overcoming diagnostic challenges. Furthermore, recent advancements in dynamic contrast-enhanced magnetic resonance imaging (DCE-MRI) techniques, have significantly enhanced the ability to track the kinetic enhancement patterns of complex breast lesions, offering higher predictive values and specificity for differentiating aggressive malignancies from benign mimics (16). A structured comparison of the MRI characteristics between these two cases provides practical guidance for radiologists evaluating CSMs. In Case 1, the MRI features were as follows: the mass showed irregular, thick-walled rim enhancement, with multiple irregular mural nodules showing marked enhancement and lobulated margins, along with progressive centripetal growth of enhancing solid tissue into the cystic cavity. DCE-MRI demonstrated a Type III (washout) time–signal intensity curve (TIC) in the solid component. These findings are highly consistent with the malignant features reported in the literature (17, 18). In contrast, the MRI features of Case 2 were: a predominantly cystic mass with a cyst wall that was thinner and smoother than that of Case 1, no discrete mural nodules, and mild-to-moderate homogeneous enhancement of the solid component; DCE-MRI showed moderate enhancement of the solid component with a Type II (plateau) TIC pattern. These observations suggest that DCE-MRI kinetic heterogeneity parameters—particularly the TIC type—serve as independent and highly discriminating predictors of malignancy in BI-RADS 4 breast lesions (11, 19, 20).

Conventional ultrasound has inherent limitations in characterizing the complex internal architecture of CSMs, often resulting in suboptimal BI-RADS categorization (21–26). In our cases, both patients initially received low-risk assessments. When initial US and mammography are insufficient to characterize a CSM, contrast-enhanced imaging modalities may provide additional diagnostic information. Contrast-Enhanced Ultrasound (CEUS) can improve detection by revealing tumor microcirculation (27), specifically centripetal heterogeneous enhancement patterns (28–31). However, its clinical adoption remains limited by equipment availability, operator dependency, and the paucity of standardized protocols. Contrast-enhanced mammography (CEM) is increasingly recognized as a more clinically accessible and cost-effective alternative: exploiting iodine-based intravenous contrast, CEM generates high-energy subtraction images that delineate enhancing lesions based on neovascularity, offering diagnostic performance broadly comparable to DCE-MRI in detecting breast malignancy while being faster, less expensive, and operable on standard mammographic platforms (32). For patients in whom MRI is contraindicated or unavailable, CEM represents a practically valuable problem-solving tool in the multimodal workup of indeterminate CSMs. While modalities like elastography offer auxiliary data, they are susceptible to interference from inflammatory fluid and should be interpreted with caution (33).

### 4.3 Pathological precision: the role of vacuum-assisted breast biopsy

If a high suspicion of malignancy persists after initial assessment with MRI (or in combination with other imaging modalities), percutaneous biopsy is then indicated. The definitive diagnosis of CSM relies on precise histopathological sampling. Case 1 highlights the risk of “sampling error” inherent in heterogeneous masses. When biopsy needles are positioned within necrotic areas or cystic cavities, only non-specific inflammatory debris is retrieved, leading to false-negative results. Relevant studies indicate that cytological examination via FNA is typically reserved for evaluating breast lesions with a low clinical suspicion of malignancy (34, 35). FNA cytology is fundamentally insufficient as a primary diagnostic tool for CSMs with suspected solid components, owing to these inherent limitations. First, FNA provides only cytological samples, which lack the architectural context necessary to differentiate complex lesions like MBC from benign mimics. Second, CSMs are inherently heterogeneous lesions, often composed of variable proportions of cystic fluid, necrotic debris, inflammatory components, and viable tumor tissue. During FNA, the needle is frequently directed into the cystic or necrotic areas, which are more easily accessible but may lack representative malignant cells. Consequently, the aspirated material may predominantly contain inflammatory cells or acellular debris, increasing the risk of false-negative or misleading cytological interpretations.

Furthermore, studies have confirmed that the VABB system significantly reduces the underestimation rate of malignancy compared to CNB (94.8% vs. 91.1%, *P* = 0.009) (36). By allowing for multi-point, large-volume sampling of the vascularized solid components, VABB effectively mitigates sampling bias, ensuring that the most aggressive components of the tumor are captured for analysis. This approach strongly aligns with the 2025 international consensus on image-guided breast biopsy, which recommends VABB as the preferred and standard technique for evaluating complex cystic and solid lesions, particularly those identified on MRI (37). Moreover, studies have shown that MRI-guided VABB can further improve diagnostic performance (38). Recent long-term cohort studies have reaffirmed that MRI-guided VABB significantly reduces the histological underestimation rate of high-risk and malignant breast lesions compared to conventional core needle biopsy, ensuring optimal surgical and oncological planning (39). Therefore, for CSMs with clinico-radiological discordance, we advocate for VABB over limited core needle biopsy (CNB) or FNA.

The diagnosis of CSM necessitates a robust “triple assessment” integrating clinical history, multi-modal imaging, and precise pathological sampling. For rapidly growing breast lesions, clinicians must look beyond superficial inflammatory presentations. Integrating DCE-MRI and ensuring representative sampling via VABB are imperative strategies to avoid misdiagnosis and ensure timely oncological intervention for rare malignancies like MBC.

## Data Availability

The original contributions presented in the study are included in the article/supplementary material. Further inquiries can be directed to the corresponding author.
